# A Review of the Appropriate Nutrition Welfare Criteria of Dairy Donkeys: Nutritional Requirements, Farm Management Requirements and Animal-Based Indicators

**DOI:** 10.3390/ani9060315

**Published:** 2019-06-01

**Authors:** Federica Raspa, Laura Cavallarin, Amy K. McLean, Domenico Bergero, Emanuela Valle

**Affiliations:** 1 Department of Veterinary Science, University of Turin, Grugliasco, 10095, Italy; domenico.bergero@unito.it (D.B.); emanuela.valle@unito.it (E.V.); 2 ISPA-CNR, Institute of Sciences of Food Production, Grugliasco, 10095, Italy; laura.cavallarin@ispa.cnr.it; 3 Animal Science, University of California Davis Davis, CA 95616, USA; acmclean@ucdavis.edu

**Keywords:** donkey, lactation, welfare, nutritional requirements, farm management requirements, animal-based indicators

## Abstract

**Simple Summary:**

The increase in dairy donkey farms in Europe, as a consequence of the increasing scientific interest in donkey milk for paediatric nutrition, has led to the need for a better understanding of the welfare of dairy donkeys. Taking into account the Animal Welfare Indicator’s (AWIN) welfare assessment protocol for donkeys, the aim of this review has been to obtain insight into good feeding welfare principles, in order to identify and discuss the nutritional requirements, the farm management requirements and the animal-based indicators that may be used to achieve an overall assessment of the appropriate nutrition welfare criteria of dairy donkeys.

**Abstract:**

Data are available in the scientific literature concerning the quality and usefulness of donkey milk for human consumption. However, there is a lack of studies related to the understanding of the welfare of dairy donkeys. The only attempt, at a European Union level, to assess the welfare of donkeys is that of the Animal Welfare Indicator’s (AWIN) welfare assessment protocol for donkeys, where the appropriate nutrition welfare criteria have been assessed, but only through the evaluation of the body condition score. However, several other indicators that take into account the importance of good feeding welfare principles should be considered for the correct management of dairy donkeys. Therefore, it is hoped that this review of the available scientific literature will be useful to help establish a set of appropriate welfare requirements and indicators for the management of dairy donkeys. The review is aimed at identifying and discussing other requirements and indicators, such as nutritional requirements, farm management requirements and animal-based indicators, which may be important for the correct assessment of the appropriate nutrition welfare criteria and to establish best practices for the feeding of dairy donkeys.

## 1. Introduction

The donkey population in Europe had been experiencing a decline over the past twenty years, due to the growing automation in agriculture, and to the depopulation of rural districts [1]. However, the European donkey population is currently increasing, due to the production of donkey milk [2]. There has been an increased interest in the use of donkey milk as a substitute food source for infants affected by cow milk protein allergy (CMPA) or multiple food intolerances [3], and it has been tested successfully in clinical studies to provide adequate nutrition and good palatability [4,5]. Generally, children who react badly to cow milk proteins can safely consume equine milk, due to its similarity in composition to human milk [6]. However, donkey milk is lower in proteins and lipids, but higher in lactose than mare milk (Table 1).

This new interest and increasing demand for donkey milk have stimulated the setting up of farms on which dairy donkeys are reared [10]. Over the past few years, some proposals have been made, at the European Union level, to evaluate the welfare of equids (including donkeys). The AWIN welfare assessment protocol for donkeys [11] is based on the four Welfare Quality^®^ principles and their welfare criteria. Among the principles, good feeding plays a crucial role for dairy donkeys, since these animals have to produce extra milk for human consumption. However, at the moment, the good feeding principle only includes the appropriate nutrition and the absence of prolonged thirst welfare criteria; these criteria are only evaluated considering three welfare indicators, that is, the body condition score, the skin tent test and water availability [11]. However, other requirements and indicators should also be taken into account to correctly evaluate the appropriate nutrition welfare criteria. Therefore, the aim of this review has been to discuss the following requirements and indicators related to the welfare of dairy donkeys:

(i) nutritional requirements (including lactation factors and energy, protein, vitamin and mineral requirements).
(ii) farm management requirements (including the provision of adequate forage, water; bedding, space, group sizes; dental care and hoof care).
(iii) animal-based indicators (including the body condition score (BCS), fatty neck score (FNS) and bodyweight (BW)).

All these requirements and indicators are discussed in order to help optimise the welfare of donkeys used for dairy production.

## 2. Nutritional Requirements

### 2.1. Lactation Factors

A precise understanding of the requirements of donkeys during lactation helps to optimise milk yields and it is important for the welfare of both the jennies and their foals. Lactation is a critical period of the breeding cycle of dairy donkeys. According to the available literature, the lactation period of dairy donkeys ranges from 180 to 350 days [6,7,12]. Foals are weaned for as long as possible to maintain milk production. Dai et al. [13] reported a variable weaning period of between 6–12 months. Considering the shape of the donkey lactation curve, the peak of the milk yield occurs at around 4–5 weeks from parturition, in a similar way to mares [14,15].

### 2.2. Energy Requirements

Currently, there is a lack of studies that establish the energy requirements of lactating donkeys. Further work is required to determine the energy requirements for milk production. However, information is available on the growth rates of donkey foals; a study conducted by De Paolo et al. [16] estimated a daily body weight (BW) gain of 0.4 kg for Martina Franca foals in the first six months (3 kg BW/week).

It has been reported that 10 L of milk are required to increase the weight of a foal by one kilogram [15], consequently, a Martina Franca foal needs around 4 L/day to obtain a gain of 3 kg BW/week. However, lactating donkeys are also milked, and have a milk yield of between 0.8 to 2 L/day [15]. It can therefore be estimated that the milk production of a Martina Franca jenny with a medium BW of 320 kg is around 4–6 L/day in the first six months of lactation. This quantity has been reported to be quite constant during the first six months [7,17], and it is therefore important to estimate the energy requirement for the first months of lactation.

The mean energy requirement per kg of milk produced by a jenny has been estimated to be around 3 MJ of digestible energy (DE), or 2.54 MJ of net energy (NE), throughout the whole lactation period [18]. Therefore, the energy requirement for the production of 5 L of milk/day would be 15 MJ of DE and 12.7 MJ of NE. The energy requirement needed for milk production should be added to the Maintenance Energy Requirement (MER). According to Smith and Burden [19], the maintenance level of a mature donkey is about 0.08–0.095 MJ of DE/kg of BW/day, considering a possible seasonal effect. From this range, it is possible to calculate a mean value of 0.09 MJ of DE/kg of BW/day, which can be considered as the MER of a moderately active adult animal in a thermoneutral environment [15]. This value is lower than the one proposed for ponies of comparable BW, which is calculated as the minimum DE requirement and estimated to be 0.13 MJ kg of BW [20].

If the requirements are evaluated according to the French Net Energy System [15], a range of 0.26–0.31 MJ of NE/kg of BW^0.75^/day may be proposed, with a mean MER of 0.29 MJ of NE/kg of BW^0.75^/day.

A comparison of the proportional increase above the maintenance energy requirements for milk production for a 300 kg BW jenny, considering a milk production of 5 L is reported in Table 2. As indicated in the table, the energy requirement during lactation increases considerably; the proportional increase of the DE requirement is 62.5% above the maintenance for the minimum, and 52.6% for the proposed elevated MER. Instead, the proportional increase above the maintenance energy requirements for the NE requirement is 67.9% for the minimum and 57% for the elevated maintenance requirements.

Because of the difference in the estimated energy requirements of the two energy systems and the lack of data, here, estimation of the energy requirement taking into account the BCS of the animal and the actual energy intake supplied with the diet is proposed. It is particularly important to perform feed analyses, since forages may have different compositions and nutritional values, according to the floristic composition and the environmental growing conditions [21]. Table 3 reports the calculation of the energy and crude protein (CP) content of diets according to the French Institut National de la Recherche Agronomique (INRA) [15] and the American National Research Council (NRC) [20], using forages of different quality. Estimating a dry matter (DM) intake of 1.6% of BW, as suggested by Burden [22], a 300 kg BW dairy donkey should eat 4.8 kg of DM/day. As shown in Table 3, the amount of energy supplied from the diet differs to a great extent on the basis of the quality of the forage. In fact, if a poor quality forage is fed, the amount of supplied energy may be reduced by half and, consequently, there may be a loss in BW. An improvement in the quality of the forage leads to an improvement in the quality of the supplied proteins. However, if this improvement is not sufficient, some authors, such as Pearson [23] and Martin-Rosset [15], have recommended using a variable amount of cereal-based concentrates, according to the nutrient value of the forage, in order to balance both the energy and the protein intake. According to the adaptation indications provided by Martin-Rosset [15], a lactating donkey should eat 3.3 kg DM/100 kg of BW as forage and 1.65 kg DM/100 kg of BW as a concentrate during the first three months of lactation. However, more studies are needed to obtain a better understanding of the DM intake of dairy donkeys and their needs.

### 2.3. Protein Requirements

Little information is available concerning the protein requirements of donkeys. Some studies have suggested that the protein requirements of donkeys may be lower than those of ponies and horses [22,24,25]. No data are available on the CP requirement of lactating donkeys, although Smith and Burden [19] determined that the maintenance CP requirement is 40 g CP/100 kg of BW/day.

INRA [15] expresses the protein requirement in Matières Azotèes Digestibles Cheval (MADC) or horse digestible protein, which represents the estimated measure of the quality of the absorbed amino acids provided by a diet [15]. The daily MADC requirement of a 300 kg Martina Franca donkey, kept at maintenance, should be around 151–166 g/day, (2.0–2.2 g MADC BW^0,75^) and 33 g MADC/kg milk during lactation [18]. Therefore, assuming a daily milk production of 5 L, it is possible to estimate that the MADC requirement for a lactating jenny is 316–331 g/day. This means that the MADC requirement for a 300 kg BW lactating jenny is roughly twice that of the normal maintenance requirement, as can be seen in Table 4.

### 2.4. Vitamin and Mineral Requirements

No specific vitamin requirements are currently known for lactating donkeys. Smith and Burden [19] suggested that the maintenance requirements of horses, as recommended by the NRC [20], may represent an appropriate amount for lactating donkeys. However, the same authors pointed out the ability of donkeys to thrive on lower vitamin and mineral levels than those recommended for horses.

It may also be important to ensure sufficient levels of vitamin supplementation, especially, for those animals that are primarily fed on dried forages [22]. Pastures may be helpful since, according to Valle et al. [9], green forages can increase the vitamin liposoluble content of milk.

The mineral requirements of lactating donkeys are unknown, and the suggestion of some authors is to use the NRC recommendations [20] concerning maintenance. Calcium and phosphorus increase noticeably, especially during the first three months of lactation. On the other hand, potassium and magnesium are usually provided with the forages.

## 3. Farm Management Requirements

Farm management requirements include different management procedures which could affect the appropriate nutrition welfare criteria on a dairy donkey farm. Therefore, this section includes those requirements (forage and water provision, appropriate bedding, adequate space and appropriate group size, optimal dental and hoof care) which are believed to play a crucial role in respecting the best practices for the feeding of donkeys.

### 3.1. Provision of Adequate Forage

In nature, donkeys are able to adapt to grazing for long periods of time, and forages are the basis of their diet [19]. There are at least three main reasons to consider forages as the basis of lactating donkey diets and for their welfare.

The first reason is that a forage-based diet is crucial for the gastrointestinal physiology of donkeys [26]. Donkeys have evolved as browsers as well as grazers. They eat low-energy, fibrous plants and spend 14–18 h per day foraging, in order to meet their nutritional requirements [27]. Donkeys may have the ability to adapt their feeding strategy to the quality and the quantity of the available feedstuff [28,29]. Compared with other equids, donkeys have shown a greater ability to use forages efficiently, even when they are of poor quality. Smith and Person [27] associated this improved digestibility with a reduced dry matter intake (DMI), but also a longer gut retention time [30]. However, it is difficult to have a clear idea regarding the DMI of donkeys. Some indications may be extrapolated from the available scientific literature. The DMI of an adult donkey fed an ad libitum forage-based diet has been estimated to be about 1.2–3.2% of the BW, depending on the type of forage and its quality (see Table 5).

The difference in the results of the various studies may be due to a difference in the voluntary dry matter intake (VDMI), which depends on the kind of feedstuff and the physiological condition of the animals. No specific recommendations are available on the minimum forage intake for either the maintenance of donkeys or for lactating donkeys but, due to their nature, forages should be given ad libitum and the body condition of the animals should be monitored to avoid overfeeding.

The second reason, as pointed out by Smith and Burden [19], is that donkeys may be sensitive to the introduction of feedstuffs with a high starch content into their diets. Moreover, sudden changes in diets could lead to gastrointestinal disorders and/or other metabolic problems such as laminitis [32]. The sensitive nature of the microbial community of a donkey makes it necessary to use diets that are based only on forages, and it discourages the use of concentrated meals [19]. Therefore, donkeys that require extra energy, as they do during lactation, could be fed hay with a higher energy content, when the available pasture forage is not sufficient on its own to maintain their body condition. Forage quality should be evaluated on the basis of the stage of maturity, since this has a profound effect on the energy and nutrient composition [33]. Moreover, although the energy content decreases with advancing maturity, it should be recalled that, according to the geographical area, the forage quality can be affected to a great extent by the grass composition and climate changes. If the forage is not sufficient to maintain the appropriate body condition of a donkey during lactation periods, extra energy should be introduced with forage-based products [22] that are available on the market. These usually have a higher energy content, since they contain some superfibres and fat.

According to Dougal et al. [34], a larger bacterial core community was found, at least for horses, for hay diets, that accounted for 15.9% of the total operational taxonomic units (OTUs), while a hay plus oil diet and hay and starch diet OTUs accounted for 10.3% and 5.4%, respectively. A smaller difference emerged in the bacterial community structure for the hay and oil diets than in the starch diets.

The third reason is related to the fact that a forage-based diet has an effect on milk quality. Few studies are available on this topic, but Valle et al. [9] reported that pasture feeding increased the fat content and fat-soluble vitamin concentration of milk. Chiofalo et al. [35] described how fresh meadow herbage vs. meadow hay feeding resulted in a higher polyunsaturated fatty acid concentration in the milk.

### 3.2. Provision of Adequate Water

The provision of water has often been overlooked in the literature. It is important to underline that, although donkeys are known for their thirst tolerance, this should not be confused with their water requirements [23]. It appears that donkeys have lower water needs than other domesticated animals [29]. For example, in a study conducted by Pearson et al. [31], it was found that ponies needed to drink more water than donkeys when both consumed the same diet. Donkeys drank 27% less water (51 mL/kg BW) than ponies (65 mL/kg BW) under the same housing and feeding conditions [36]. An interesting feature of donkeys is that the feed intake of water-deprived donkeys only decreased by 10%, while it decreased by 30% for ponies under the same conditions [19].

The amount of water a donkey needs depends on several factors: diet (fresh forages versus hay-based diets), work, physiological status and environment temperature. Lactation leads to an increase in water intake in all milk-producing animals, because the losses due to lactation represent one of the major ways by which water is lost [36]. Lactating donkeys need about twice the amount of water as non-producing donkeys [23]. The general rule to ensure the welfare of dairy donkeys is to provide donkeys with free, unlimited access to fresh, clean water [29]. However, donkeys are particularly sensitive to the temperature of water. According to Smith and Burden [19], it is necessary to provide water at a higher temperature than 15 °C, since they may refuse to drink cold water. An inadequate water intake could cause such gastrointestinal problems as constipation and colic [19]. In this context, it is advisable to introduce buckets with warm water during winter. It should also be considered that when water is supplied by automatic drinkers, it is important to control the functioning and flow rate. Nyman and Dahlborn [37] showed that horses had a greater water intake when the flow rate was 8 L/min.

### 3.3. Provision of Appropriate Bedding

The type of bedding material that is used for donkeys may be of concern for their appropriate nutrition welfare criteria, because edible bedding materials may represent a risk factor for the development of nutritional diseases. The choice and quantity of the bedding material and the bedding management (regularly changed and/or cleaned) are important factors [11,38], especially when dairy donkeys are housed in intensive farming systems where the animals are kept in stalls, without access to pastures. A study conducted by Burden et al. [39] showed that the use of chopped cardboard and paper used as bedding for donkeys could enhance the risk of hyperlipemia, due to the fact that donkeys seem to prefer to eat cardboard and paper to any available fresh haylage and straw. Cox et al. [40] recognised that the use of paper bedding increased the risk of colic. Even though many veterinarians and owners are discouraged from feeding straw to equids, in order to avoid colic and gastric ulcers, in the above-cited studies there was no evidence of this risk when donkeys were bedded on straw. Barley straw is reported to be the safest bedding for donkeys to ingest [41]. However, it is necessary to pay attention to straw bedding, if it is being consumed: straw can still have grains that could enhance energy and starch intake, thus leading to overweight donkeys and the risk of laminitis [19].

Even though the intention of this section is to underline the possible risks of using specific bedding that could be ingested, it is also necessary to evaluate the hygienic quality of the bedding. The use of appropriate bedding material and its absorption capacity have been shown to be important in equine management to preserve the health of both the respiratory tract and of the hooves [42,43,44].

### 3.4. Provision of Adequate Space and Appropriate Group Sizes

No studies are available on the adequate space and appropriate group sizes of dairy donkeys. However, this aspect could represent an important issue that should be considered for the appropriate nutrition welfare criteria of dairy donkeys. Donkeys need to receive an appropriate diet in order to maintain their milk production and right body condition, and for this reason, an appropriate group size should be organised to avoid the risk of underfeeding or overfeeding some of the animals. The design of the groups should take into account the natural structure of a donkey herd. However, few studies are available on this topic. According to McGreevy [45], ecological factors, such as the abundance and distribution of food and water, have been reported to influence the social organisation of donkeys. Donkeys usually tend to form pair bonds within a herd [45], and the only stable groups in feral environments have been described to be those of females and their offspring.

Minimising negative social interactions and maintaining the stability of the herd may also be important factors. Moving donkeys from one group to another may have social implications that could lead to an unstable social environment. Consequently, keeping well-adapted dairy donkeys together may help to contribute towards a stable inner structure of the group. This, in turn, would promote the welfare of dairy donkeys, since a stable social interaction and psychological bond would be maintained. In this way, it is possible for the farmer to have more control over the feeding intake of the animals and to adjust the diet according to their productivity.

Little is known about the group sizes of dairy donkeys. The group size may have an important effect on the feeding behaviour and feed intake of donkeys. Avoiding overcrowding could also be important for dairy donkeys, because it has been shown that aggressive behaviour between donkeys might be reduced when the social groups live in an environment with an abundance of accessible resources for all [45,46]. The number of animals within a group should be decided on in relation to the available space. It is essential to guarantee sufficient space for all the donkeys in order to allow them to express their natural lying and moving behaviour and to reduce competition for available resources. The AWIN welfare assessment protocol for donkeys [11] indicates that a lack of space raises competition for individual space within the herd, and this can enhance stress, which in turn affects the temperament of the animals. According to the same protocol, a donkey with a wither height of less than 120 cm needs a shelter area of 5.5 m^2^, while one with a height of between 120–148 cm needs an area of 7 m^2^. However, this protocol refers just to the shelter dimensions, and no mention is made of the dimensions of the areas where they move (such as pastures or the dry lot areas where the donkeys are kept). Moreover, no mention is made of the bunk length or feeding space. These features may be important, especially for donkeys kept on semi-extensive breeding farms where access to pasture is limited. Semi-extensive farms are characterised by a combination of both intensive and extensive husbandry methods. Dairy donkeys are partially grazed and partially fed ad libitum on these farms with on-farm produced hay [8,9], as a consequence of the impossibility of satisfying their requirements with pasture forages alone.

The accessibility of each group member to feeds is important to maintain the well being of the animals and the correct VDMI.

### 3.5. Provision of Optimal Dental Care

Dental care is one of the most important farm management indicators of appropriate nutrition. In a study conducted on lactating donkeys, the researchers noticed that animals with a lower BCS (a score of 1 or 2 scores out of 5) showed poorer dental conditions [47]. In addition, some studies have shown that dental disorders in donkeys are associated with weight loss [48,49] and the possible onset of intestinal diseases [50]. Donkeys are monogastric animals and, consequently, have a more simplified digestion system than ruminants, which means that they should have the opportunity to mechanically breakdown feeds to a particle size [19]. One of the most common health issues of equids is due to the presence of untreated uneven teeth; this condition influences the donkey’s chewing behaviour and, consequently, affects the intake and digestion of their feeds [28,29], due to sharp points and hooks on the molars. An impaired dental function and/or dental pain might lead to a higher risk of colic and to weight loss, as a consequence of a reduction in the daily energy intake [39]. A dental examination of donkeys should be focused on how the animals chew. In this regard, Valle et al. [47] proposed a scoring system to monitor the ability of donkeys to chew. Dental disorders in donkeys have been shown to be one of the major and most frequently underestimated problems for the welfare of donkeys [25] and are often the least recognised and/or treated [51]. Rodrigues et al. [51] found that most donkeys with documented dental disorders showed few to no signs of pain. Therefore, since it may be difficult for an owner or practitioners to recognise these conditions, it is important to subject the animals to regular and scheduled dental checks. An oral examination should be conducted at least once a year, by a qualified equine dental professional, so that they receive appropriate treatment, if needed [26].

### 3.6. Provision of Optimal Hoof Care

There is an old saying that states “no foot, no horse”, and this is an important point as an indicator of the appropriate nutrition welfare criteria of donkeys. The pain associated with hoof disorders could lead to changes in feeding behaviour, such as a reduction in feed intake and in the number of daily visits to the feeder. No studies on the feeding behaviour of dairy donkeys, as a consequence of hoof problems, are currently available. However, it has been reported that hoof pain in horses and donkeys can lead to an increase in periods spent lying and to a lack of movement [52]. It is known that the correct management of donkey hooves, with regular and programmed care, is one of the principal ways of preventing painful diseases, such as lameness and laminitis [53]. In a study conducted in a number of facilities in Italy and the United Kingdom, 15.16% of the assessed donkeys presented overgrown hooves and evidence of incorrect hoof trimming [54]. As a consequence, it is important that owners and producers are informed about the correct shape of the hoof and the need for regular care to improve the welfare of donkeys. Hoof quality depends not only on regular trimming but also on nutrition and on the environment where the donkeys are kept. The link between laminitis and nutrition has long been recognised: feeding equines with high-energy amounts of concentrate feedstuff has been associated with the onset of overweight, gastrointestinal disorders and laminitis [55].

## 4. Animal-Based Indicators

The animal-based indicators of donkeys are evaluated directly on the animals and may mirror such effects as under and/or overfeeding and metabolic diseases. Moreover, they may be used as nutritional and management indicators.

### 4.1. Body Condition Score (BCS)

BCS is the only indicator considered in the AWIN welfare assessment protocol for the appropriate nutrition welfare criteria for donkeys [11]. BCS is an important welfare indicator, because it is the only one that shows whether the energy requirements have been fulfilled [56]. It is necessary to minimise the loss of body weight during the first period of lactation. For this reason, it is important to reach foaling with a BCS of 3.5–4 according to the Smith and Burden five-point scale [19], since the BCS of jennies could decrease by 0.5–1 point during the first months of lactation, even if they receive a balanced diet. However, the BCS indicator may be considered an imprecise tool as the scores are based on subjective interpretations. Valle et al. [47] reported that when an untrained person, such as a dairy donkey breeder, assigns a BCS score, there is a risk he/she may inflate the score of a thin animal on the basis of only the abdomen conformation. However, it has been documented that BCS is very useful when performed with specific protocols [56]. In this context, two BCS scoring systems have been developed to classify donkeys according to their physical appearance and to the presence of adipose tissue in key areas. Pearson and Ouassat [57] described the key areas of the donkey’s body that should be considered during a BCS assessment: neck, wither, shoulders, point of shoulders, spinous process of the vertebral column, ribs, flank, hooks or tuber coxae, rump and pins or tuber ischii. Table 6 shows a comparison of the two scale systems used on adult donkeys. The Pearson and Ouassat scoring system [57] gives marks on a nine-point scale: 1—emaciated, 2—thin, 3—less thin, 4—less than moderate, 5—moderate, 6—more than moderate, 7—less fat, 8—fat, 9—obese. The other scale, provided by the Donkey Sanctuary [22], evaluates donkeys on a five-point scale: 1—poor, 2—moderate, 3—ideal, 4—fat, 5—obese; it also allows half points to be given if the donkey is at an intermediate level. However, both scales have problems in recognising small changes in the body condition [58]. For this reason, it is important to develop standardised methods to detect these small changes in the subcutaneous fat covering, in part because fat deposits in donkeys may be unevenly distributed, especially over the neck and hindquarters [22]. Moreover, fat stores can remain when there is a loss in the overall weight and/or may calcify [26]. Consequently, each anatomical area should be evaluated and combined carefully to obtain an overall condition score. Other limitations are related to the fact that BCS should not be considered as the only appropriate nutrition welfare indicator, since it may also be affected by dental treatments [47] and parasite infestations [59].

### 4.2. Fatty Neck Score (FNS)

In recent years, there has been an increase in the number of studies related to the regional adiposities of horses and ponies. Particular attention was paid to the adipose tissue deposited along the crest of the neck, which has been associated with the development of metabolic problems, such as insulin resistance and an enhanced risk of laminitis [60,61]. Several studies have shown that donkeys also frequently develop a fatty crest, which tends to droop on one side [19]. Smith and Burden [19] recognised that the development of such a fatty crest is a clear indication of donkey fattening.

Valle et al. [47] developed a scoring system for donkeys, named “fatty neck score” (FNS) (Table 7), which involves the assessment of the morphometric measurement of the neck thickness. As shown in Table 7, the score is similar to that developed for horses by Carter et al. [61]. However, this scale attempts to make this indicator more objective through the measurement of the neck thickness of the donkey. This score system was tested, by the same authors, on dairy donkeys, and it was reported to be significantly correlated to BCS [47]. However, FNS should be evaluated in dairy donkeys, since it can be independent of the overall adiposity status, because a certain level of regional adiposity may be maintained even when the overall bodyweight decreases [22,26]. In this context, it is important to evaluate and monitor BCS and FNS together in order to perform a correct evaluation of the nutritional status of lactating donkeys, although more studies are necessary to understand the possible implication between FNS and metabolic diseases.

### 4.3. Body Weight (BW)

The best way to evaluate the nutritional status of dairy donkeys is to monitor BCS and BW together [22,57]. Although the use of weighing scales is the best way of determining the BW of donkeys, they are difficult to use in field conditions. Thus, the estimation of the BW through body measurements may be considered an acceptable tool for BW estimation [62]. Equations have been developed to estimate the BW of adult donkeys using different body measurements and coefficients. The following measurements should be taken with a soft tape, while ensuring an adequate tension: the height at the withers; the heart girth taken around the body, caudal to the elbow (olecranon tuber), two centimetres behind the highest point of the withers; the body length from the elbow to the pin bone (tuber ischia). In order to improve the accuracy of these measurements, it is essential that they are taken when the donkey is standing on level ground.

Pearson and Ouassat [57] developed an equation to estimate the BW (1) of an adult donkey with a BCS of 2 to 6 on the nine-point scale and a height of 90–120 cm, which involves measuring the heart girth and the body length.

Live weight (kg) = (heart girth [cm]^2.12^ × length [cm]^0.688^)/3801 (1)

The same authors also developed an Equation (2) to estimate the BW of adult donkeys with a BCS > 6 [57].

Live weight (kg) = (heart girth [cm]^2.575^) × (height [cm]^0.240^)/3968 (2)

The Donkey Sanctuary [22] developed an Equation (3) to estimate the BW of adult donkeys using the height at the withers and heart girth measurements.

Live weight (kg) = 0.000252 × height at the wither [cm]^0.24^ × heart girth [cm]^2.575^ (3)

Pearson and Ouassat [57] proposed an Equation (4) to estimate the BW of donkeys using only the heart girth measurement. This method is suitable for handling restless or intractable donkeys, especially if no other person is available to help restrain the animal.

Live weight (kg) = heart girth [cm]^2.65^/2188 (4)

Nomograms are also available for the estimation of the BW. In this case, a line is drawn on the chart to link the heart girth to the body length measurements [57] or the heart girth to the height at the withers [38]. The point where the line crosses the weight line on the nomogram indicates the weight of the donkey.

## 5. Conclusions

This review summarises the nutritional requirements, farm management requirements and animal-based indicators that should be considered when evaluating the appropriate nutrition welfare criteria of dairy donkeys. Furthermore, this review explores the connections between animal-based indicators and appropriate nutrition welfare criteria. The evaluation of the good feeding welfare principle remains multi-faceted and complex. A wide range of requirements and indicators should be considered when assessing the welfare of lactating donkeys. The scarcity of information in the current literature highlights the need for further studies on the requirements and indicators necessary to optimise the welfare of these animals. A robust evaluation should take into account an integrated set of indicators, instead of just a single one. 

## Figures and Tables

**Table 1:** Chemical composition (g/100 g) of donkey and mare milk.

Milk Source	Lipids	Proteins	Lactose	Ash
Donkey	0.3–1.8 ^1^	1.3–2 ^2,3^	6.4–7.9 ^3^	0.3–0.5 ^1^
Horse	0.5–2.0 ^1^	1.5–2.8 ^1^	5.8–7.0 ^1^	0.3–0.5 ^1^

^1^ Data adapted from Guo et al. [7]. ^2^ Data adapted from Cavallarin et al. [8]. ^3^ Data adapted from Valle et al. [9].

**Table 2:** Estimated digestible energy (DE) and net energy (NE) maintenance requirements for a lactating donkey (with a bodyweight (BW) of 300 kg and assuming a daily milk production of 5 L) with the proportional increase over the maintenance requirements (adapted from Smith and Burden [19] and Martin-Rosset [18]).

Requirements	Digestible Energy, DE		Net Energy, NE	
	Minimum	Elevated	Minimum	Elevated
MER ^1^ (MJ)	24 ^2^	28.5 ^2^	18.7 ^3^	22.3 ^3^
Energy requirement (MJ) per 5 kg of produced milk	15		12.7	
Total energy requirement (MJ) (MER ^3^ + Energy for milk production)	39	43.5	31.4	35
Proportional increase above the maintenance requirements (%)	62.5	52.6	67.9	57

^1^ MER: maintenance energy requirement; ^2^ Data adapted from Smith and Burden [19]; ^3^ Data adapted from Martin-Rosset [18].

**Table 3:** Theoretical diets considering different forage qualities for a dairy donkey (300 kg BW) during the first three months of lactation *.

Forage Quality	Energy Provided by the Forage	Energy Provided According to the DM Forage Intake
According to INRA	NE ^1^	NE ^1^
Good quality, first-cut hay	5.4	25.8
Poor quality, first-cut hay	3.2	15.4
According to NRC	DE ^2^	DE ^2^
Good quality, first-cut hay	8.4	40.2
Poor quality, first-cut hay	4.2	20.1

^1^ NE: net energy (MJ/kg of DM); ^2^ DE: digestible energy (MJ/kg of DM). * Considering a dry matter (DM) intake of 1.6% of BW/day (4.8 kg of DM).

**Table 4:** Daily MADC requirements for a 300 kg BW lactating donkey according to INRA ^1^.

Requirements	MADC
Maintenance requirement	144.2–158.6 g
Total requirement per 5 kg of milk	165 g
Total requirement (maintenance+milk)	309.2–323.6 g
Proportional increase above the maintenance requirement	114.4–104%

INRA: Institut National de la Recherche Agronomique. MADC: Matières Azotèes Digestibles Cheval.

**Table 5:** Comparison of the dry matter intake (DMI) of different forages expressed in g/kg of BW^0.75^ and percentage DMI/100 kg BW.

Forages	DMI (g/kg BW^0.75^)	% DMI	Number of Donkeys
Meadow hay ad libitum ^1^	81 ^1^	2.6	4 ^1^
Barley straw ad libitum ^1^	37 ^1^	1.2	4 ^1^
Alfalfa hay ad libitum ^1,2^	100 ^1,2^	3.2	5 ^1,2^
Oat straw ad libitum ^1,2^	60 ^1,2^	1.9	4 ^1,2^

^1^ Data adapted from Wood [24]; ^2^ Data adapted from Pearson et al. [31].

**Table 6:** Comparison of the two body condition scoring systems for donkeys. Adapted from Pearson and Ouassat [57] and The Donkey Sanctuary [22]. The images in the table are the authors’ own.

Body Condition Scoring for Donkeys				
Scale From 1 to 9		Scale From 1 to 5		
1. Very thin (Emaciated)	Bone structure easily seen over body. Little muscle present.	1. Poor	Neck thin and it meets the shoulders abruptly. Shoulders angular. All bones easily felt. Dorsal spine of the withers and backbone prominent. Dorsal and transverse processes easily felt. Ribs can be seen from a distance and felt easily. Belly tucked up. Little muscle covering on the hindquarters. There may be a cavity under the tail.	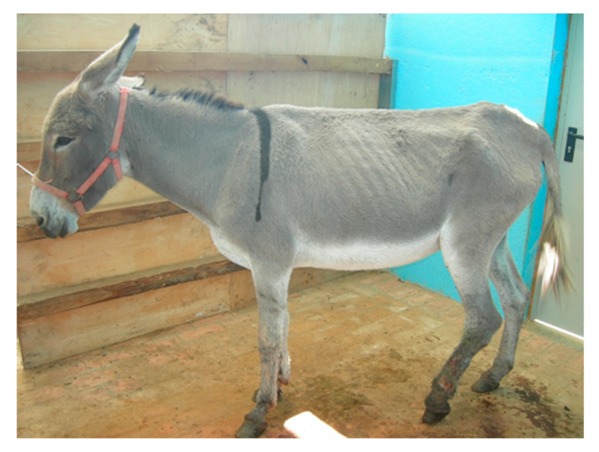
2. Thin	Bone structures are prominent and sharply defined. Neck thin and shoulders sharply angular. Some muscle development.			
3. Less thin	Little muscle or fat covering the bone structures, which can be felt easily. Loin area and rump concave.	2. Moderate	Some muscle development overlying the bones, which can be felt easily. Slight step on neck-shoulder junction. Some covering over the dorsal withers. Spinous processes may be felt but are not prominent. Ribs, dorsal/transverse processes and hips not visible, but can be felt easily.	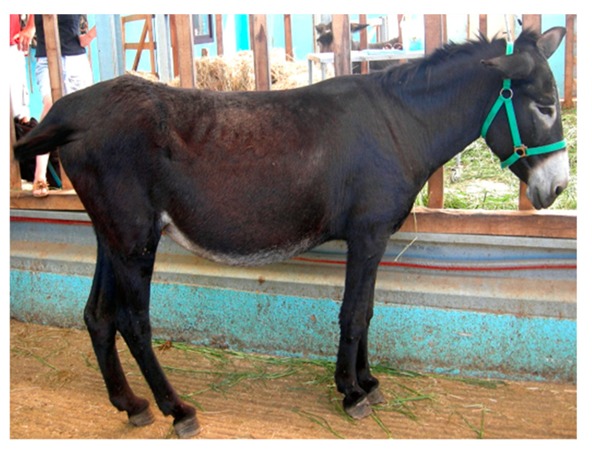
4. Less than moderate	Vertebral column visible. Withers, shoulders and neck have some muscle and fat covering. Pins can be felt but not visible. Hooks rounded but visible. Rump flat rather than concave.			
5. Moderate	Hooks and rump rounded. Pins not visible. Some fat can be felt in the shoulder area and at the base of the neck. Ribs may be felt, but not visible.	3. Ideal	Good muscle development. Bones felt under light covering of the muscle/fat. Neck flows smoothly into the shoulders, which are rounded. Ribs just covered by a slight layer of fat/muscle. Ribs can be felt. Spinous and transverse processes cannot be felt. Hip bones rounded in appearance, and can be felt	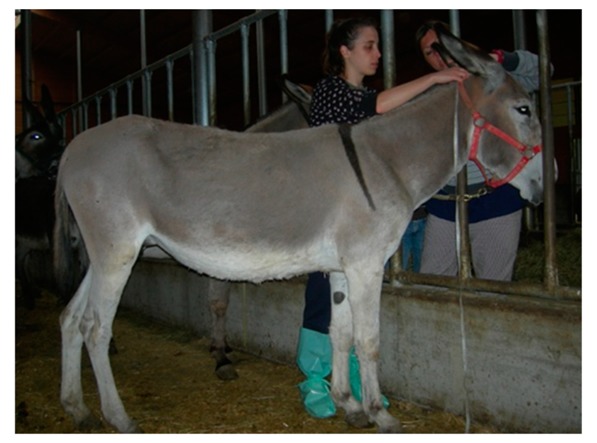
6. More than moderate	Spinous processes cannot be felt easily. Rump convex and well-muscled. Some fat can be felt on the neck.	4. Fat	Neck thick. Crest hard. Shoulders covered in an even fat layer. Withers broad, bone felt with firm pressure. Dorsal ribs only felt with firm pressure; ventral ribs may be felt more easily. Bone structure can only be felt with firm pressure. Belly overdeveloped.	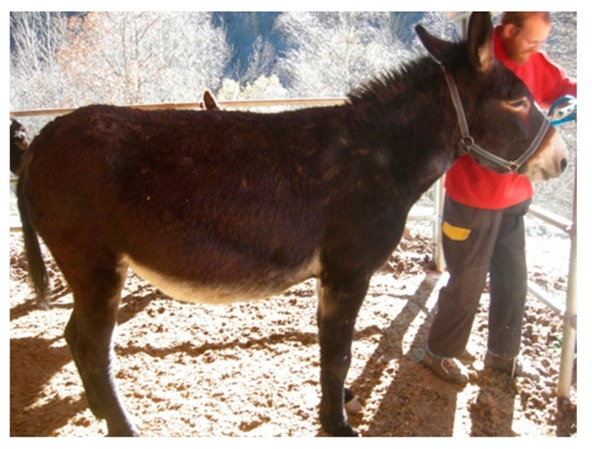
7. Less fat	Spinous processes cannot be felt. Hooks just visible. Fat on neck and shoulders is beginning to expand over the rick. Neck thickening.			
8. Fat	Animal appears well covered and with a rounded body but no fat or bones are discernible. Flanks filled, broad back.	5. Obese	Neck thick, crest bulging with fat and falling to one side. Shoulders bulging with fat. Withers broad. Bone structures cannot be felt. Ribs not palpable. Belly pendulous in depth and width. Back broad. Spinous and transverse processes cannot be felt. Deep crease along midline and bulging fat either side.	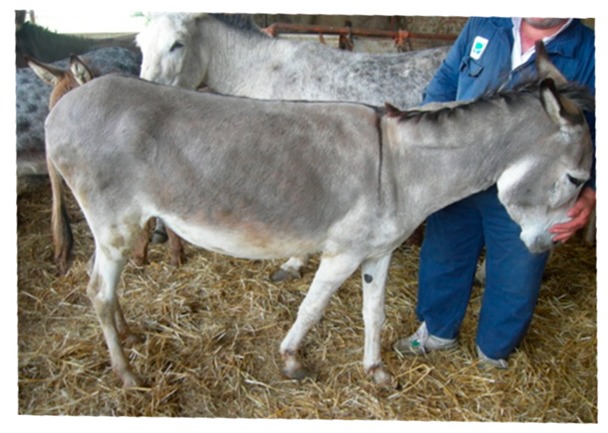
9. Very fat	Bones buried in fat. Large accumulations of fat on the neck, over the shoulder area and on the ribs. Flank filled with fat.			

**Table 7:** Fatty neck score (FNS) for donkeys. From Valle et al. [47].

Score	Illustration of the Individual Fatty Neck Score	Description	Neck Thickness Range According to FNS (in cm)
0	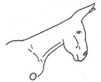	Neck: thin with the absence of a visible and palpable crest.	<14
1	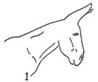	Neck: thin with no visible crest, but a slight filling felt upon palpation.	>14–19
2	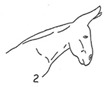	Neck: with a moderate deposition of fat. Noticeable appearance of a crest, with fat deposited fairly evenly from the poll to the withers. Crest: easily cupped in one hand and easily bent from side to side.	>19–22
3	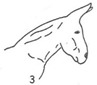	Neck: enlarged and thickened. Crest: palpable from the poll to the withers, filling a cupped hand, and beginning to form longitudinal fat deposits on both sides of the neck.	>22–27
4	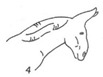	Neck: very enlarged and thickened. Crest: grossly thickened with fat deposits from the poll to the withers, forming longitudinal bands of fat on both sides of the neck. Crest cannot be bent easily from side to side.	>27–34
5	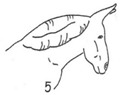	Neck: very enlarged and thickened. Crest: very thickened with hard fat deposits, rounded along both sides of the neck.	>34
